# Collective Honesty? Experimental Evidence on the Effectiveness of Honesty Nudging for Teams

**DOI:** 10.3389/fpsyg.2021.684755

**Published:** 2021-07-08

**Authors:** Yuri Dunaiev, Menusch Khadjavi

**Affiliations:** ^1^Independent Researcher, Frankfurt, Germany; ^2^Department of Spatial Economics, School of Business and Economics, Vrije Universiteit Amsterdam, Amsterdam, Netherlands; ^3^Tinbergen Institute, Amsterdam, Netherlands; ^4^Kiel Institute for the World Economy, Kiel, Germany

**Keywords:** honesty, lying, nudge, team, experiment

## Abstract

A growing literature in economics studies ethical behavior and honesty, as it is imperative for functioning societies in a world of incomplete information and contracts. A majority of studies found more pronounced dishonesty among teams compared to individuals. Scholars identified certain nudges as effective and cost-neutral measures to curb individuals' dishonesty, yet little is known about the effectiveness of such nudges for teams. We replicate a seminal nudge treatment effect, signing on the top of a reporting form vs. no signature, with individuals and confirm the original nudge treatment effect. We further ran the same experiment with teams of two that have to make a joint reporting decision. Our results show the effectiveness of the nudge for teams, which provides further confidence in the applicability of the nudge.

## Introduction

The subject of dishonesty and deception is undergoing intense study and arouses high concerns in the society, attracting much attention of policymakers and researchers from the fields of behavioral economics and psychology (e.g., Rosenbaum et al., 2014; Abeler et al., 2019; Gerlach et al., 2019; Köbis et al., 2019). Beyond ethical considerations, the economic harm caused by dishonesty is tremendous. The Association of Certified Examiners estimates that the typical firm losses are about 5% of revenues to occupational fraud each year, which translates into a loss of $3.6 billion at the global level (ACFE, 2020). Recent examples show that practices such as manipulation of financial and audit reports and fraudulent accounting methods are a major problem. Among convicted companies are big names such as Enron, Lehman Brothers, Madoff Investment Securities, and Parmalat. Other famous fraudulent practices are spying (Hewlett-Packard), violations of safety regulations (Southwest Airlines), and concealing emission levels (Volkswagen). In all of these fraud cases it was not a single individual who made the decision and guarded misconduct from coming to light, but teams of individuals who deceived in a conspirative manner.

Since Thaler and Sunstein (2009) introduced the concept of nudging to a larger audience, a number of experiments from psychology and economics have shown that certain nudges can work to reduce *individual* dishonesty (e.g., Mazar et al., 2008; Shu et al., 2012; Fellner et al., 2013,^1^). A related literature on individual vs. team (dis)honesty developed contemporaneously and suggests that teams are often more dishonest than individuals (e.g., Cohen et al., 2009; Sutter, 2009; Danilov et al., 2013; Mühlheußer et al., 2015; Weisel and Shalvi, 2015; Korbel, 2017; Wouda et al., 2017; Kocher et al., 2018; Dannenberg and Khachatryan, 2020)^2^. The mechanisms that cause teams to be more dishonest include greater sophistication regarding the consequences of lying (Cohen et al., 2009; Sutter, 2009) and diffusion of responsibility regarding the moral misconduct of lying (Kocher et al., 2018)^3^.

As dishonesty levels and mechanisms differ between individuals and teams, we regard it as a natural question whether nudges that are able to curb individual dishonesty remain effective for teams. In this paper we answer this question by employing the well-established math puzzle task paradigm and honesty nudge of Shu et al. (2012)^4^. To this end, we test whether we are able to replicate one of the treatment effects of Shu et al. (2012)—asking decision makers to sign that they will report honestly at the top of a reporting form compared to a no-signature control treatment. We ran the experiment for individuals and for teams to test for the robustness of this nudge.

Our experiment indeed successfully replicates the treatment effect of Shu et al. (2012) for individuals, adding further evidence that signing on top of the form can decrease dishonesty (compared to the no signature condition). For teams we find the same treatment effect, which shows further robustness of this nudge. The nudge seems to be able to work against the team dishonesty drivers like the diffusion of responsibility. We regard our finding as good news for policy makers who seek to employ such nudges as a tool for low-cost and effective anti-fraud and anti-corruption measures.

This paper proceeds as follows. In second section we provide the details of the experimental design, hypotheses and procedures. Third section presents the results and fourth section concludes.

## Experimental Design

In this section we explain the details of the math puzzle (or matrix) task and the treatments we employed. We subsequently relate our treatments to hypotheses that originate from the current literature on lying of individuals and teams and finally provide information about the procedures of the experiment.

**Figure 1:**
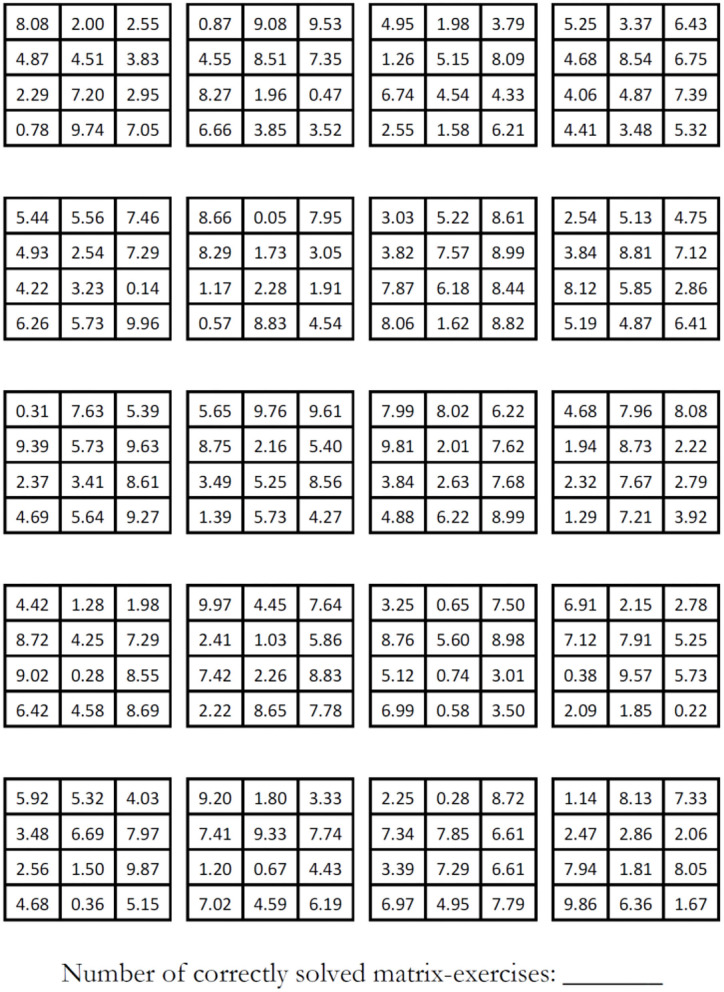
A complete math puzzle sheet (original is in A4 format).

The math puzzle (matrix) task comprised sheets of paper with math puzzles (matrices) where two numbers sum exactly to a specific target number that is defined beforehand. In the case of Shu et al. (2012) and our experiment, each puzzle consisted of 12 three-digit numbers (with two decimal digits) of which two numbers sum exactly to the number 10. The task was to identify these two numbers and circle them in order to “solve” the respective puzzle. Each correctly solved puzzle yielded a piece-rate income, in our experiment 0.50 EUR. In the treatments with individuals (teams) we provided one (two) sheets of paper, with 20 puzzles per sheet of paper. Hence, a maximum of 10 EUR could be earned per participant in this task. Teammates could choose to work on each sheet separately or together. The time limit was strictly set to 5 min and stopped with a stop-clock. We calibrated the time limit to ensure that the solved puzzles are well-distributed between 0 and 20. Participants were asked to sum the score at the bottom of the puzzle sheet. Figure 1 shows a complete sheet as used in our experiment.

If the number of correctly solved puzzles (or matrix exercises), i.e., the true score, is common knowledge, then it is straightforward for the researcher who conducts the experiment to multiply this score with 0.50 EUR and pay out the individual or team accordingly. If the true score is private knowledge of the individual or team, then it becomes interesting to investigate under which circumstances there is correct or elevated reporting of the true score.

In order to create a scenario in which participants would feel comfortable to over-report their score, we closely followed the procedure of Shu et al. (2011)—a study by three of the five authors of Shu et al. (2012) whose treatment effect we aim to replicate. We asked participants to dispose of the matrix paper sheet by inserting it into a paper shredder. The shredder was prepared in a way that the sheet would be partly shredded at the sides, but remain intact to retrace the scores. This incomplete shredding was not visible to participants, as the sheets moved through the shredder into a non-transparent bin. Note that for this replication approach we followed procedures of Shu et al. (2011) closely, which falls into a gray area of omitted information as categorized by Charness et al. (2021). While the scenario is suggestive of sheets being destroyed, we neither commented on sheets being destroyed nor did we indicate that we would not have a look at sheets after the sessions. This gave us the chance to learn the true score of all individuals and teams after the sessions and link them to the reported scores.

**Figure 2:**
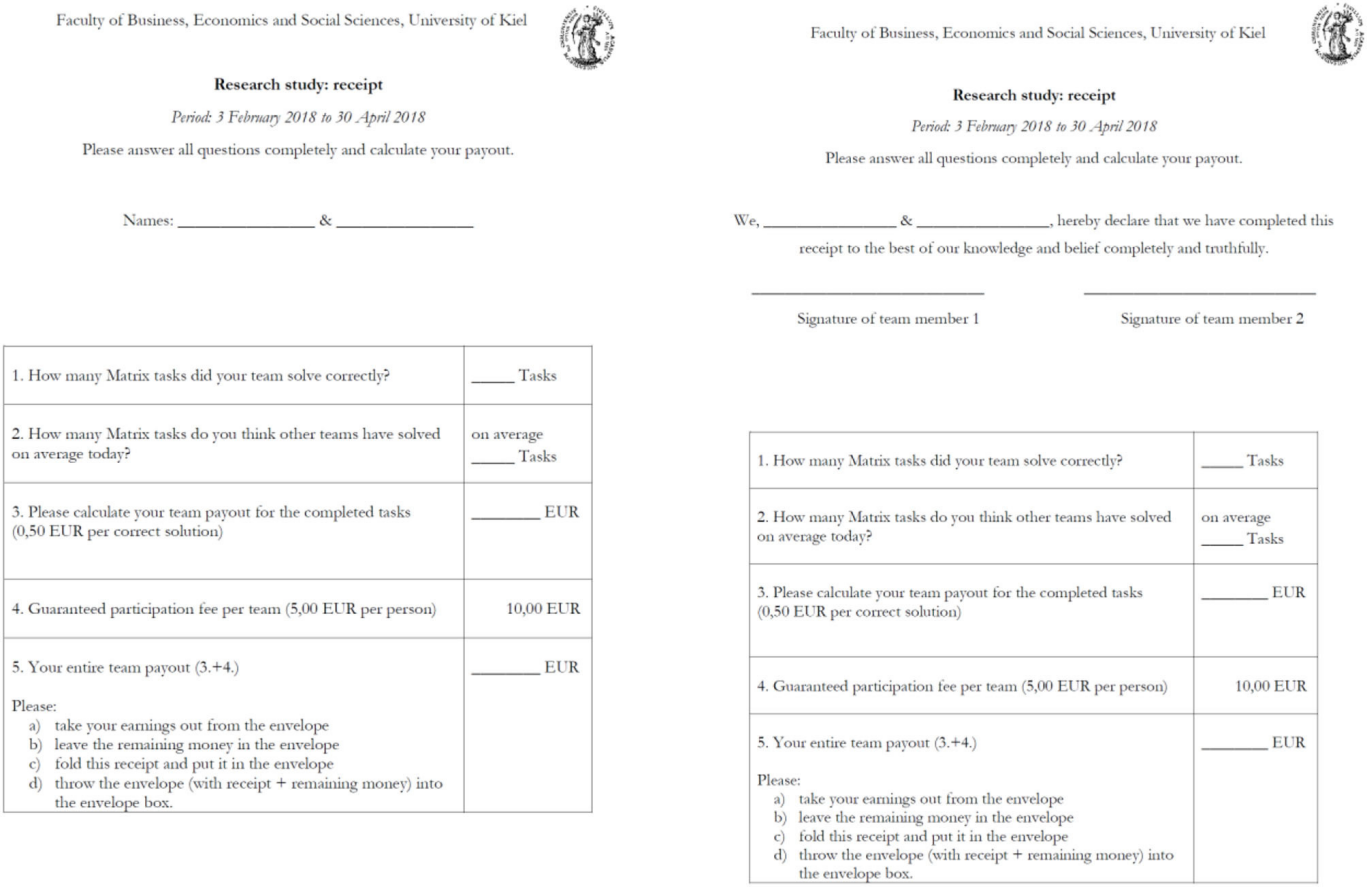
The receipt forms in team treatments. Appendix 2, 3 provide the receipt forms in a larger resolution.

For score reporting we used the participation receipt (see Figure 2). The receipt included reporting the score, guessing the average score of others in the session (not incentivized), multiplying the score with 0.50 EUR and adding a 5-EUR show-up fee per person. It is on this receipt that the individuals or teams could misreport their scores. Receipt forms for the respective treatments were handed to the participants after they had completed the matrix task. All individuals and teams had envelopes at their desk with 15 EUR (individuals) or 30 EUR (teams) in cash, so that any payment dividable by 0.50 EUR was possible. Subsequently, they took their payments out of the envelopes, folded and inserted the receipts into their envelopes, kept their cash payment and dropped the envelopes with the receipts, and unclaimed cash into a return box.

The receipt forms in all treatments included a line (lines) to provide the name of the individual (names of teammates). The difference between the no-signature and signature treatments consisted of the following additional statement at the top of the receipt form that the participants in the signature treatments: “We, [line(s) for name(s)], hereby declare that I (we) have completed this receipt to the best of my (our) knowledge and belief completely and truthfully.” Participants in the signature treatments had to sign underneath the statement. Note that there were no instructions or information that suggested any form of detection or punishment related to the statement.

Shu et al. (2012) introduced an honesty nudge which is able to decrease dishonesty and fraud of individuals—signing on the top of a form compared to no signature. They suggested that this nudge helps to turn to an individual's morality and to promote honesty right before the deception may take place—in our experiment before potentially over-reporting the score.

Literature on the dishonesty of teams often points into the direction that teams are more prone to lying than individuals (Danilov et al., 2013; Mühlheußer et al., 2015; Weisel and Shalvi, 2015; Korbel, 2017; Wouda et al., 2017; Kocher et al., 2018; Dannenberg and Khachatryan, 2020). Teams tend to me more strategic about lying and deception (Cohen et al., 2009; Sutter, 2009) and diffusion of responsibility and moral disutility appear to be key drivers (Kocher et al., 2018). Given that these mechanisms appear to promote dishonesty of teams, it is questionable whether the signature honesty nudge remains effective for teams. If it does, it would be good news for practitioners who employ pledges with signatures to curb dishonesty—yet if the nudge treatment effect is limited to individuals, it would greatly reduce the usefulness of the nudge and potentially other similar nudges, as many fraudulent situations actually involve teams of decision makers. Table 1 provides an overview of our treatments.

**Table 1:** Treatment cells.

		**Moral commitment**	
		**No signature**	**Signature on top**
Decision maker composition	Individual	Ind_NOsig	Ind_sig
	Team	Team_NOsig	Team_sig

Based on the literature described above, we therefore formulate our key hypothesis that over-reporting of scores is lower in the *_sig* treatments compared to *_NOsig* treatments—both when comparing individuals' reporting decisions and teams' reporting decisions. Hence, we hypothesize that the nudge is effective for teams despite possible counteracting effects from diffusion of responsibility. In order to proceed with a testing our hypothesis, it was essential to replicate finding of Shu et al. (2012) for individual decision makers in our environment and conditions. A total of 127 students of the University of Kiel were recruited through the hroot platform (Bock et al., 2014) and participated in the experiment in the time period February to April 2018. There were 20 and 23 participants in *Ind_NOsig* and *Ind_sig* treatments, respectively. In the *Team_NOsig* and *Team_sig* treatments there were 42 participants per treatment, yielding 21 independent team observations per treatment^5^. The teams were formed randomly by participants of a session drawing numbers on balls from a non-transparent bag.

Following the literature on team dishonesty (e.g. Sutter, 2009), communication between team members may be important to let them get to know each other, develop intra-team trust, exchange thoughts on the task and on motivation to (mis)report the effort. For this reason, we implemented our experiment in a way that team members sat together in a large cubicle. Hence, face-to-face communication of team members was possible throughout the session.

In order to facilitate the team feeling even more, we implemented an additional stage using a creativity task before the actual matrix task and reporting^6^. This task was included in order to help teammates to get to know each other a bit better and “break the ice.” Allowing communication when completing tasks together was supposed to mimic situations when teams are working and making decisions together in the real environment. In the creativity task individuals (in the *Ind_* treatments) and teams (in the *Team_* treatments) were given 10 min to create a picture of their choice by using a whiteboard and pins of different colors (see Appendix 4 for an example). The instructions explicitly informed the participants that there were no incentives related to their creativity or performance and that they were free to do whatever they like. Note that all individuals and teams created a picture, even though an empty whiteboard would have been just as acceptable. In order to be consistent, participants in the *Ind_* treatments also performed this task, but alone. After this creativity task, we ran the matrix task describe above.

## Results

**Figure 3:**
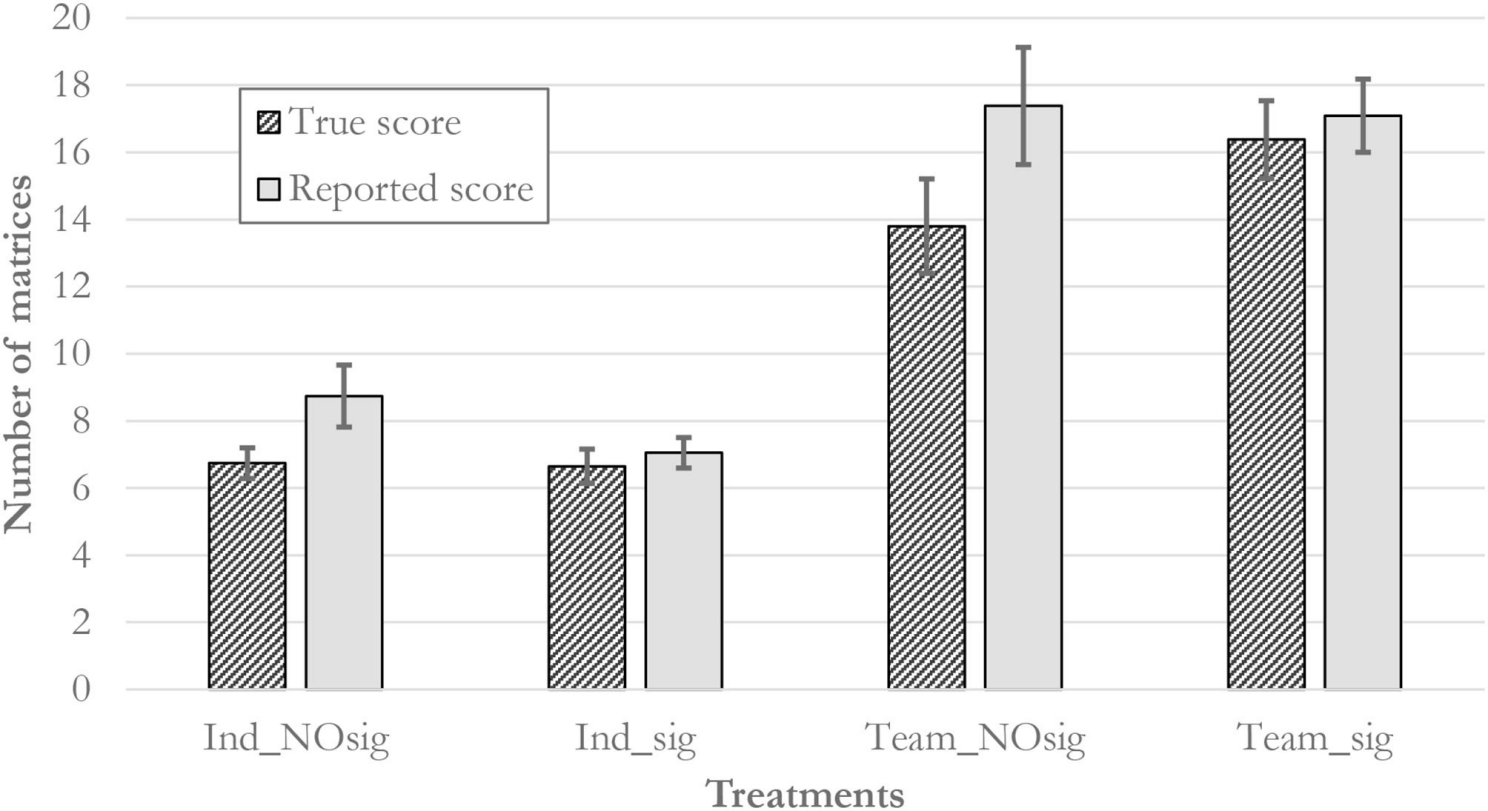
Mean true and reported scores in the four treatments. The bars depict ±1 standard error.

Table 2 provides summary statistics of our treatments and Figure 3 provides an overview of mean reported as well as actually solved matrices. For the following analysis we compare the reported number of solved matrices with the number of solved matrices as noted down on the matrix sheet (see bottom of Figure 1) to detect willful dishonesty. We begin this section with an examination of the *Ind_* treatments in order to see whether our results confirm the treatment effect of Shu et al. (2012). In *Ind_sig* fewer individuals over-reported (10%, 2 out of 20) as compared to *Ind_NOsig* (39%, 9 out of 23), which is different based on a (two-sided) Fisher's exact test (*p* = 0.039). Employing Wilcoxon signed-rank tests for differences between score summaries and claimed scores in the receipt for each individual, we find that there is significant over-reporting in *Ind_NOsig* (8.74 reported matrices vs. 6.91 summarized matrices, *p* = 0.0039) and no detectable over-reporting in *Ind_sig* (7.05 vs. 6.80, *p* = 0.500). We therefore find strong support that including the signature nudge at the top of the receipt form reduces dishonesty significantly. Hence, we replicate Shu et al. (2012)'s result (signature on top vs. no signature) for individual decision makers.

**Table 2:** Descriptive statistics.

**Metric**	**Treatments**			
	***Ind_NOsig***	***Ind_sig***	***Team_NOsig***	***Team_sig***
Mean solved matrices, as checked by researchers	6.74	6.65	13.81	16.38
Mean solved matrices, as summarized on the matrix sheet	6.91	6.80	14.19	17.24
Mean matrices tried (marked with circles)	7.30	6.80	15.09	17.38
Mean reported matrices in the receipt form	8.74	7.05	17.38	17.09
Guess of mean solved matrices of others	8.35	7.15	14.95	16.71
Share willfully lying	39.1%	10.0%	33.3%	4.7%
Number of participants	23	20	42	42
Number of independent observations	23	20	21	21

We proceed with a similar analysis for the *Team_* treatments to detect whether the signature nudge remains effective in this scenario. Indeed, we find that there are 7 out of 21 teams (33.3%) that over-report their scores on the receipts in *Team_NOsig* compared to only 1 out of 21 (4.7%) in *Team_sig*. These propensities are, again, significantly different from each other (two-sided Fisher's exact test, *p* = 0.045). Wilcoxon signed-rank tests confirm that there is detectable over-reporting in *Team_NOsig* (17.38 matrices claimed vs. 14.19 matrices summarized as solved, *p* = 0.0156), there is no detectable different in *Team_sig* (17.09 vs. 17.24, *p* = 0.9725)^7^. We therefore find clear evidence that the signature nudge curbs dishonesty of teams effectively, alike the scenario for individuals. The result does not support a claim that teams' dishonesty is qualitatively different in a way that makes teams immune to this nudge.

## Conclusion

This paper asked whether moral nudges that work to curb dishonesty of individuals also remain effective for teams—units that are ubiquitous in companies and have been shown to act more sophisticatedly and feel less responsible for their actions as the outcome of the team's decision rests on the shoulders of several team members (Falk and Szech, 2013; Kocher et al., 2018; Falk et al., 2020). We employ the seminal finding of Shu et al. (2012) who showed that asking for a signature to confirm honesty at the top of a form fosters honesty compared to no signature. The main argument is that this can help to turn to an individual's morality and promote honesty exactly before misreporting may take place.

After the successful replication of Shu et al. (2012)'s effect for individuals, we extended the finding by confirming that this nudge is equally effective for a team setting, resulting in an 86% decrease in the amount of cheating teams. In our eyes, the presented research makes an important contribution to a better understanding of team behavior and in developing instruments for preventing teams and individuals from deception and cheating.

To the best of our knowledge, this is the first study to investigate the effectiveness of moral nudges for teams and it should be considered as a starting point for avenue of future research. Future research may investigate the dimensions of familiarity of team members, which our creativity task aimed for, further. Likewise, our teams consisted of two members and future research could vary this dimension by examining behavior of larger teams. Field experimental methods could be used decrease scrutiny of laboratory experiments and similar studies with higher stakes could check for the robustness of our and Shu et al.'s findings. Such investigations seem promising to test the ecological validity of our results. We regard as highly policy-relevant to investigate team decision-making and develop cost-effective instruments like nudges that can be implemented in practice by organizations and policymakers to curb fraud and dishonesty of teams.

## Data Availability Statement

The raw data supporting the conclusions of this article will be made available by the authors, without undue reservation.

## Ethics Statement

Ethical review and approval was not required for the study on human participants in accordance with the local legislation and institutional requirements. Written informed consent for participation was not required for this study in accordance with the national legislation and the institutional requirements.

## Author Contributions

YD wrote his master thesis under the supervision of MK and this work is a concise product coming out of this collaboration. YD and MK developed the research question and the experiment material together. YD and MK ran the experiment together and YD analyzed the data. MK contributed the creativity task as a team-building exercise, wrote the paper, and financed the experiment. All authors contributed to the article and approved the submitted version.

## Conflict of Interest

The authors declare that the research was conducted in the absence of any commercial or financial relationships that could be construed as a potential conflict of interest.

## Footnotes

^1^Note that there is a replication discussion around Mazar et al. (2008): see also Amir et al. (2018) and Verschuere et al. (2018). Verschuere et al. (2018) report one of the results of Mazar et al. (2008) does not replicate based on a meta-analysis with more than 5,000 participants. Amir et al. (2018) reply to Verschuere et al. (2018) and discuss conceptual challenges with direct replication studies.

^2^There is also a broader literature that compares economic decisions of individuals and teams, e.g., Bornstein et al. (2004), Charness and Sutter (2012), and Kugler et al. (2012).

^3^Regarding the diffusion of responsibility and ethical behavior, see also Falk and Szech (2013) and Falk et al. (2020).

^4^There are several treatments in Shu et al. (2012): Note that Kristal et al. (2020) report that the top-vs.-bottom-signature treatment effect of Shu et al. (2012) does *not* replicate for individuals. This is not the treatment effect we aim to replicate in this paper—we concentrate on the top-signature versus no-signature comparison. In the task participants need to find two numbers in a 4 × 3 table that sum to a specific number. In Shu et al. (2012), Mazar et al. (2008) and in our experiment this number is 10.

^5^See Appendix 1 for instructions.

^6^See Kachelmeier et al. (2008); Erat and Gneezy (2016, 2017); Charness and Grieco (2019); Grözinger et al. (2020), and Kachelmeier and Williamson (2010) for economic experiments on creativity.

^7^In *Team_sig* there was even one team that reported a lower number than summarized on the matrix sheet, yet indeed the correct number when comparing the reported number of matrices with the correctly solved number checked by the research team.
