# Supplementary material for: Collective Honesty? Experimental Evidence on the Effectiveness of Honesty Nudging for Teams
Source: Front Psychol. 2021 Jul 8;12:684755. doi: 10.3389/fpsyg.2021.684755 (PMC8295523; doi:10.3389/fpsyg.2021.684755)

Collective Honesty?
Experimental Evidence on the Effectiveness of Honesty Nudging for Teams

Yuri Dunaiev and Menusch Khadjavi[1]

May 25, 2021

-Appendices below-

[1] Correspondence: Menusch Khadjavi, Vrije Universiteit Amsterdam, Department of Spatial Economics, School of Business and Economics, De Boelelaan 1105, 1081 HV Amsterdam, The Netherlands; e-mail: m.khadjavipour@vu.nl. Affiliations: Yuri Dunaiev: EB-Sustainable Investment Management. Menusch Khadjavi: Department of Spatial Economics, School of Business and Economics, Vrije Universiteit Amsterdam, Netherlands; Tinbergen Institute, Amsterdam, Netherlands; Kiel Institute for the World Economy, Kiel, Germany.

Appendix 1. General Instructions for Teams (translated from German).

General instructions

Welcome!

You are taking part in a scientific economic study. You have the opportunity to earn a certain amount of money that will depend on your decisions. It is important that you read the following explanations carefully.

The instructions, that you have received from us, are for your private familiarization only and must be returned after the study. **The communication between teams during the study is strictly forbidden. Silent communication within the teams is expressly allowed.** If you have any questions, please raise your hand and experimenter will come to you.

At this point, we would like to ask you to put your personal belongings in your bag or jacket and put them away. Please be aware that your mobile phone and any other electronic devices must be completely switched off and removed. Failure to comply with these rules will result in exclusion from the study and all payments.

Your decisions in the study must be anonymous. The teammate will be aware of your decisions, but other teams should not know what decisions you make. You will receive a guaranteed show-up fee of 5.00 EUR per person. The additional payout will depend on your decisions.

Today's session consists of **two parts**. Both parts are **independent of each other**. Your decisions will affect your payout only in **Part 2**. Decisions in Part 1 does not affect your payout.

The earnings will be paid out in cash after completing both parts. In the end, we will hand out a questionnaire and ask you to answer all the questions completely and quietly.

Instructions for Part 1

Your team task in Part 1 is to make a picture using the materials available. You have exactly 10 minutes to complete this task. Each team receives a white pegboard and different colored mushroom nails. Please handle the materials carefully. Team members **are allowed to talk quietly** to each other during the task.

In the end, we ask each team to give the picture a **title**, which you should write on the given yellow paper. The title should consist of exactly **one German word** that describes the picture. We will collect the boxes with pictures back after the time is over.

As a reminder, your payout in this study does **not** depend on completing this task.

Instructions for Part 2

In Part 2, each team receives two A4 sheets each consisting of 20 matrices. These sheets are in the envelope which is placed on your desk. You are allowed to take the sheets out of the envelope only when the time starts.

Each matrix contains 12 numbers. Your task is to find two numbers in each matrix that exactly **add up to 10**. You have 5 minutes to complete this task. For each correctly solved matrix task, your team earns 0.50 EUR, in addition to the 5.00 EUR participation fee. An example:

2.71	9.21	6.54
1.24	8.83	3.66
5.95	1.07	4.86
4.49	4.80	7.29

In the example, 2.71 and 7.29 add up to 10. Please mark the solutions for each matrix by **circling the correct numbers** (as shown above). There is not a specific order in which you need to solve the matrix tasks. Team members **can talk quietly** to each other and decide for themselves how they want to carry out the task.

As a reminder, your payout in this study depends on completing this Part.

Appendix 2. Report Form No Signature for Teams (translated from German).

Faculty of Business, Economics and Social Sciences, University of Kiel

Research study: receipt

Period: 3 February 2018 to 30 April 2018

Please answer all questions completely and calculate your payout.

Names: _________________ & _________________

1. How many Matrix tasks did your team solve correctly?	_____ Tasks
2. How many Matrix tasks do you think other teams have solved on average today?	on average<br>_____ Tasks
3. Please calculate your team payout for the completed tasks (0,50 EUR per correct solution)	________ EUR
4. Guaranteed participation fee per team (5,00 EUR per person)	10,00 EUR
5. Your entire team payout (3.+4.)<br><br>Please:<br>a) take your earnings out from the envelope<br>b) leave the remaining money in the envelope<br>c) fold this receipt and put it in the envelope<br>d) throw the envelope (with receipt + remaining money) into the envelope box.	________ EUR

Appendix 3. Report Form with Signature for Teams (translated from German).

Faculty of Business, Economics and Social Sciences, University of Kiel

Research study: receipt

Period: 3 February 2018 to 30 April 2018

Please answer all questions completely and calculate your payout.

We, _________________ & _________________, hereby declare that we have completed this receipt to the best of our knowledge and belief completely and truthfully.

______________________________ ______________________________

Signature of team member 1 Signature of team member 2

1. How many Matrix tasks did your team solve correctly?	_____ Tasks
2. How many Matrix tasks do you think other teams have solved on average today?	on average<br>_____ Tasks
3. Please calculate your team payout for the completed tasks (0,50 EUR per correct solution)	________ EUR
4. Guaranteed participation fee per team (5,00 EUR per person)	10,00 EUR
5. Your entire team payout (3.+4.)<br><br>Please:<br>a) take your earnings out from the envelope<br>b) leave the remaining money in the envelope<br>c) fold this receipt and put it in the envelope<br>d) throw the envelope (with receipt + remaining money) into the envelope box.	________ EUR

Appendix 4. Example of a Creativity Task Outcome.

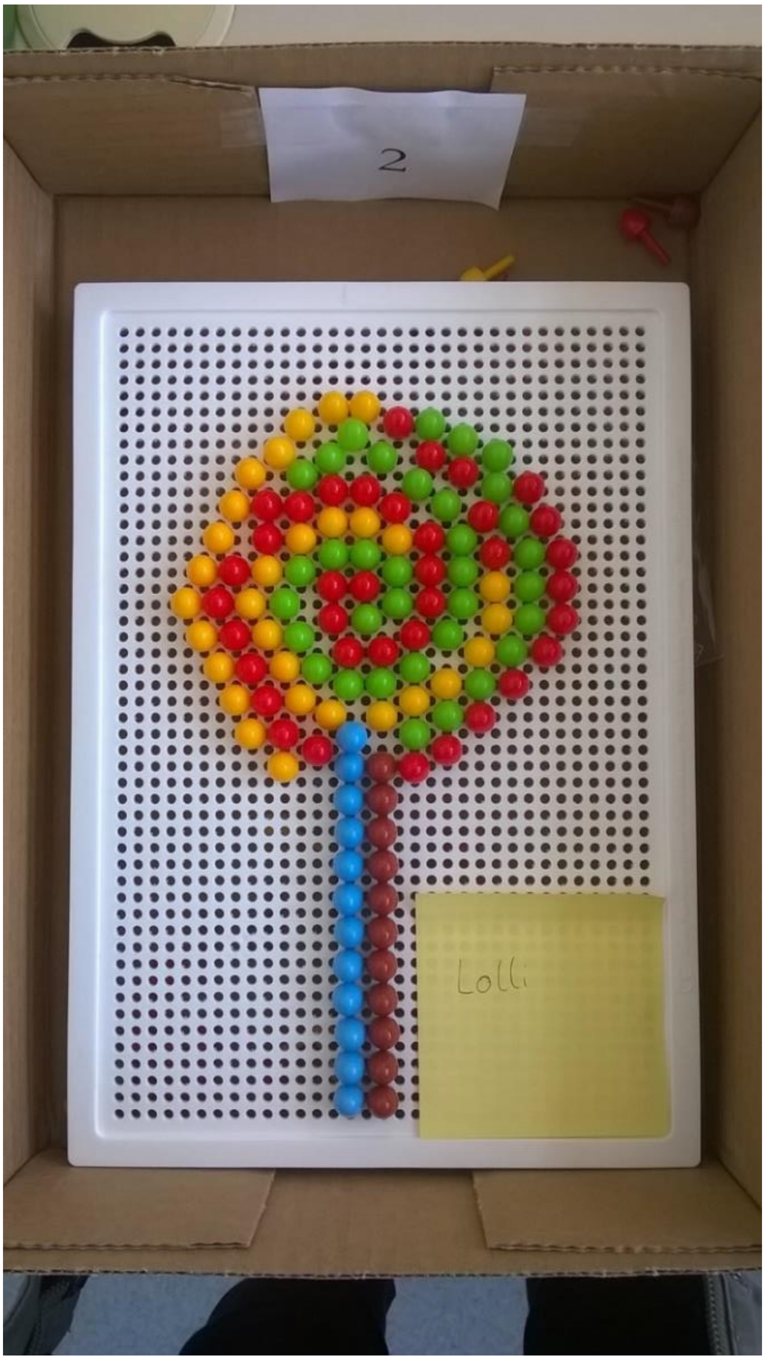

Supplement: Supplementary file 1 [file Presentation_1.pdf]
